# Paroxysmal neurological spells in TANGO2 deficiency disorder: a case report and a scoping review

**DOI:** 10.3389/fped.2026.1786640

**Published:** 2026-04-02

**Authors:** Emanuela Claudia Turco, Giulia Pisanò, Silvia Carestiato, Laura Caiazza, Benedetta Piccolo, Carlo Fusco, Susanna Esposito, Maria Carmela Pera

**Affiliations:** 1 Child Neuropsychiatric Unit, Maternal and Child Health Department, Parma University-Hospital, Parma, Italy; 2 Department of Biomedical, Metabolic and Neural Sciences, University of Modena and Reggio Emilia, Modena, Italy; 3 Department of Neurosciences Rita LeviMontalcini, University of Turin, Turin, Italy; 4 Child Neurology and Psychiatry Unit, Department of Pediatrics, Presidio Ospedaliero Santa Maria Nuova, Reggio Emilia, Italy; 5 Pediatric Clinic, Departement of Medicine and Surgery, University of Parma, Parma, Italy; 6 Child Neuropsychiatry Unit, Department of Medicine and Surgery, University of Parma, Parma, Italy

**Keywords:** dystonia, episodic ataxia, non-epileptic events, paroxysmal episodes, TANGO2 deficiency disorder

## Abstract

**Background:**

TANGO2 deficiency disorder (TDD) is a rare autosomal recessive condition characterized by neurodevelopmental impairment and recurrent metabolic crises. Paroxysmal non-epileptic neurological episodes (“TANGO2 spells”) are increasingly recognized but remain poorly characterized and often misdiagnosed.

**Methods:**

We report a child with TDD presenting with recurrent paroxysmal neurological episodes and performed a scoping review of Scopus and PubMed-indexed literature to summarize clinical features, triggers, diagnostic findings and management of TANGO2 spells.

**Results:**

The reported patient developed recurrent stereotyped episodes from 23 months of age, characterized by weakness, dystonia with head tilt, vomiting, irritability and reduced responsiveness, lasting several hours and triggered by illness or reduced intake. EEG during spells was without epileptic discharges, metabolic investigations and QTc were normal, and whole-exome sequencing confirmed biallelic pathogenic TANGO2 variants. Following supportive supplementation, no further spells or metabolic crises occurred. Across 7 studies including 93 patients identified by the scoping review, TANGO2 spells typically began in infancy or early childhood, were stress-related, transient, and self-limited, with non-ictal EEG findings when assessed. Cardiac involvement was consistently associated with metabolic crises rather than isolated spells.

**Conclusions:**

TANGO2 spells represent a distinctive early neurological manifestation of TDD that may occur independently of metabolic decompensation. Their recognition can facilitate earlier diagnosis and prompt initiation of preventive supportive strategies.

## 1 Introduction

TANGO2 deficiency disorder (TDD; OMIM #616878) is a rare autosomal recessive condition caused by biallelic pathogenic variants in the *TANGO2* gene. Clinically, TDD is characterized by global developmental delay, epilepsy, movement disorders, hypothyroidism, and recurrent episodes of metabolic decompensation, often accompanied by rhabdomyolysis and life-threatening cardiac arrhythmias (1–5). Although the molecular function of TANGO2 is not fully elucidated, accumulating evidence suggests a role in cellular stress responses and mitochondrial homeostasis (1–4).

Among the earliest neurological manifestations of TDD, a distinctive but still poorly characterized phenomenon has been described: paroxysmal neurological episodes commonly referred to as “TANGO2 spells”. These episodes are typically precipitated by physiological stressors, such as intercurrent illness, fasting, or metabolic imbalance, and have been reported in up to two-thirds of affected individuals (6). Clinically, TANGO2 spells are characterized by a transient worsening of baseline neurological function, including dystonia, abnormal head or body posture, gait impairment, dysarthria, drooling, lethargy, and reduced responsiveness. Importantly, electroencephalographic recordings obtained during these episodes are frequently without epileptiform discharges, contributing to diagnostic uncertainty and frequent misclassification as epileptic seizures or other paroxysmal neurological disorders (1, 2, 6, 7).

Although TANGO2 spells may precede or occur independently of metabolic crises, their clinical significance remains incompletely understood. The heterogeneity of reported semiology, the absence of standardized diagnostic criteria, and the overlap with epileptic and non-epileptic paroxysmal movement disorders contribute to delayed recognition of TDD and missed opportunities for anticipatory management (6, 7). Most available data derive from isolated case reports, small case series, or observational cohorts, resulting in fragmented and inconsistently reported descriptions of these episodes.

Recent experimental findings have further supported a link between TANGO2 deficiency and stress-induced cellular vulnerability. In particular, the interaction between TANGO2 and the small heat shock protein CRYAB has been implicated in cytoskeletal integrity and mitochondrial resilience, providing a potential mechanistic framework for transient neurological deterioration under metabolic stress (8). In parallel, increasing clinical recognition of TANGO2 spells as recurrent, self-limited neurological events distinct from overt metabolic crises highlights a significant gap in the phenotypic characterization of TDD (5).

A structured synthesis of how TANGO2 spells are described, triggered, investigated, and managed in the existing literature is therefore needed to improve clinical recognition and refine early diagnostic pathways.

The aims of this study are:

1. to describe the clinical presentation, diagnostic evaluation, and longitudinal outcome of a child with TANGO2 deficiency disorder presenting with recurrent paroxysmal non-epileptic neurological episodes; and
2. to conduct a scoping review of the PubMed-indexed literature to systematically map the reported clinical features, triggering factors, diagnostic findings, and management strategies associated with TANGO2 spells.

By integrating an illustrative case with a structured overview of the existing literature, this study seeks to better delineate the clinical phenotype of TANGO2 spells and to emphasize their relevance as an early diagnostic clue in TANGO2 deficiency disorder.

## 2 Methods

### 2.1 Study design

This study consists of a detailed case report combined with a brief scoping review of the literature. The scoping review was conducted to map how paroxysmal neurological episodes, commonly referred to as “TANGO2 spells”, have been described, investigated, and managed in patients with TANGO2 deficiency disorder. The review methodology was informed by the principles of the PRISMA Extension for Scoping Reviews (PRISMA-ScR).

### 2.2 Literature search strategy

A systematic literature search was performed in PubMed (MEDLINE) and Scopus from database inception to 22 February 2026. The search strategy combined free-text terms related to TANGO2 deficiency disorder and paroxysmal neurological manifestations. The following search string was applied, adapted as needed to database-specific syntax: (TANGO2 OR “TANGO2 deficiency” OR “TANGO2 deficiency disorder” OR TRMEA OR metabolic encephalomyopathic crises) AND (spell* OR paroxysmal OR episodic OR dystonia OR dyskinesia OR ataxia OR “movement disorder” OR “head tilt” OR torticollis) No restrictions were applied regarding patient age, publication date, or study design.

### 2.3 Eligibility criteria

Studies were eligible for inclusion if they met the following criteria:

1. Reported patients with genetically confirmed TANGO2 deficiency disorder;
2. Described paroxysmal neurological episodes consistent with TANGO2 spells or equivalent terminology.

Case reports, case series, and observational studies were included. Studies exclusively focused on molecular mechanisms or animal models without clinical data were excluded.

### 2.4 Study selection

Titles and abstracts were independently screened by two authors (GP and LC). Full texts of potentially eligible studies were subsequently assessed by the same reviewers. Discrepancies were resolved by discussion and consensus, without the need for a third reviewer. The study selection process is summarized in a PRISMA-ScR flow diagram.

### 2.5 Data charting and synthesis

Data were extracted using a predefined charting form. Extracted variables included: terminology used to describe paroxysmal episodes, age at onset, clinical semiology, triggering factors, electroencephalographic findings, association with metabolic crises, and reported management strategies. Given the heterogeneity of study designs and outcomes, results were synthesized descriptively and narratively without quantitative pooling.

### 2.6 Case presentation

Clinical data were collected as part of routine diagnostic evaluation and follow-up at a tertiary child neuropsychiatry center. Clinical history, neurological examinations, neurophysiological studies, neuroimaging, metabolic investigations, and genetic testing were reviewed retrospectively from medical records.

Neurological assessment included serial standardized developmental evaluations and detailed characterization of paroxysmal neurological episodes, with particular attention to age at onset, semiology, duration, triggering factors, frequency, and post-episode recovery. Electroencephalographic findings were used to support the classification of episodes. Brain magnetic resonance imaging (MRI) was performed using age-appropriate protocols.

Cardiac evaluation included electrocardiography with measurement of the corrected QT interval. Laboratory investigations comprised creatine phosphokinase, liver enzymes, and a comprehensive metabolic workup, including blood lactate, ammonia, plasma amino acids, acylcarnitine profile, urine organic acids, very-long-chain fatty acids, glucose, and cerebrospinal fluid neurotransmitters, according to standard clinical protocols.

Genetic testing was performed using family-based whole-exome sequencing. Identified variants were interpreted according to the American College of Medical Genetics and Genomics (ACMG) criteria and confirmed by segregation analysis when appropriate. Therapeutic interventions and clinical outcomes were documented during longitudinal follow-up.

### 2.7 Ethical considerations

The case report was conducted in accordance with the Declaration of Helsinki. Written informed consent was obtained from the patient's parents for publication of clinical data and images. Ethical approval was not required for the literature review component of the study.

## 3 Results

### 3.1 Case presentation

The patient is a 5-year-old male, second child of non-consanguineous healthy parents, born at term by elective cesarean section after an uneventful pregnancy. Birth weight was 2,685 g (small for gestational age), length 48 cm, and head circumference 34 cm. Apgar scores were 9 and 10 at 1 and 5 min, respectively.

During infancy, the clinical course was characterized by gastroesophageal reflux disease, poor appetite, irritability, and frequent nocturnal awakenings. Developmental delay became evident during the second year of life.

From the age of 23 months, the child developed recurrent, stereotyped paroxysmal neurological episodes consistent with TANGO2 spells. Episodes were characterized by sudden onset of muscular weakness associated with dystonic posturing, torticollis with abnormal head tilt, vomiting, irritability, and reduced responsiveness, without loss of consciousness. Episodes typically lasted several hours and resolved spontaneously, followed by marked fatigue and prolonged recovery. The frequency increased during periods of intercurrent illness or reduced oral intake, suggesting a clear association with physiological stress and fasting. No fixed circadian pattern was observed.

Importantly, no biochemical evidence of metabolic decompensation was documented during or immediately after these episodes.

Interictal electroencephalography showed background slowing with superimposed fast rhythms in the bitemporal regions and occasional sharp waves. These findings were considered mildly abnormal for age but nonspecific. Importantly, no ictal discharges or electroclinical correlates were observed during paroxysmal episodes, supporting their non-epileptic nature. Brain MRI revealed bilateral temporopolar arachnoid cysts with ventriculomegaly and increased extra-axial cerebrospinal fluid (CSF) spaces These findings were considered incidental, as no progressive structural abnormalities or acute lesions were identified, and no clear association with the clinical phenotype could be established. Cardiac evaluation, including electrocardiography, demonstrated a normal corrected QT interval.

Baseline laboratory investigations, including creatine phosphokinase, liver enzymes, and an extensive metabolic workup (blood lactate, ammonia, plasma amino acids, acylcarnitine profile, urine organic acids, very-long-chain fatty acids, glucose, and cerebrospinal fluid neurotransmitters), were within normal limits.

Array-based comparative genomic hybridization was normal. Family-based whole-exome sequencing identified compound heterozygous pathogenic variants in *TANGO2*, consisting of a paternal deletion of approximately 34 kb involving exons 3–9 and a maternal c.473C > T variant, confirming the diagnosis of TANGO2 deficiency disorder.

Based on the paroxysmal presentation, differential diagnoses included epilepsy, GLUT1 deficiency, *ADCY5*-related dyskinesia, and idiopathic paroxysmal dyskinesias. Epilepsy was excluded due to the absence of ictal EEG activity and the prolonged, stereotyped, non-epileptic nature of the episodes.

Supportive therapy was initiated, including coenzyme Q10, B-complex vitamins (B1, B5, B9), vitamin D3 and L-carnitine. Following treatment initiation, no further paroxysmal neurological episodes consistent with TANGO2 spells were reported.

At the age of 5 years and 6 months, the child ambulates independently and has acquired limited expressive language. Formal cognitive testing using the non-verbal Leiter-3 scale could not be completed due to poor cooperation. Adaptive functioning was formally assessed using the Vineland Adaptive Behavior Scales, Second Edition (Vineland-II), Survey Interview Form, completed by the patient's mother.

The Adaptive Behavior Composite yielded a standard score of 62, consistent with a globally low level of adaptive functioning (<1st percentile for age). Domain-specific standard scores indicated low adaptive functioning in Communication (SS = 55), Daily Living Skills (SS = 68), and Motor Skills (SS = 69), and a moderately low level in Socialization (SS = 81).

Age-equivalent scores revealed marked delays across communication subdomains (receptive, expressive, and written skills ranging from 2 years 10 months to 3 years 7 months). Daily living skills were globally impaired, with the exception of domestic skills, which were age-appropriate. In the socialization domain, interpersonal relationships and coping skills were moderately delayed, while play and leisure skills were more markedly affected. Motor skills showed a dissociation, with low fine motor performance and relatively preserved gross motor abilities.

Overall, the assessment documented clinically significant adaptive impairments across multiple domains, with relative strengths in domestic daily living skills and gross motor functioning.

**Figure 1:**
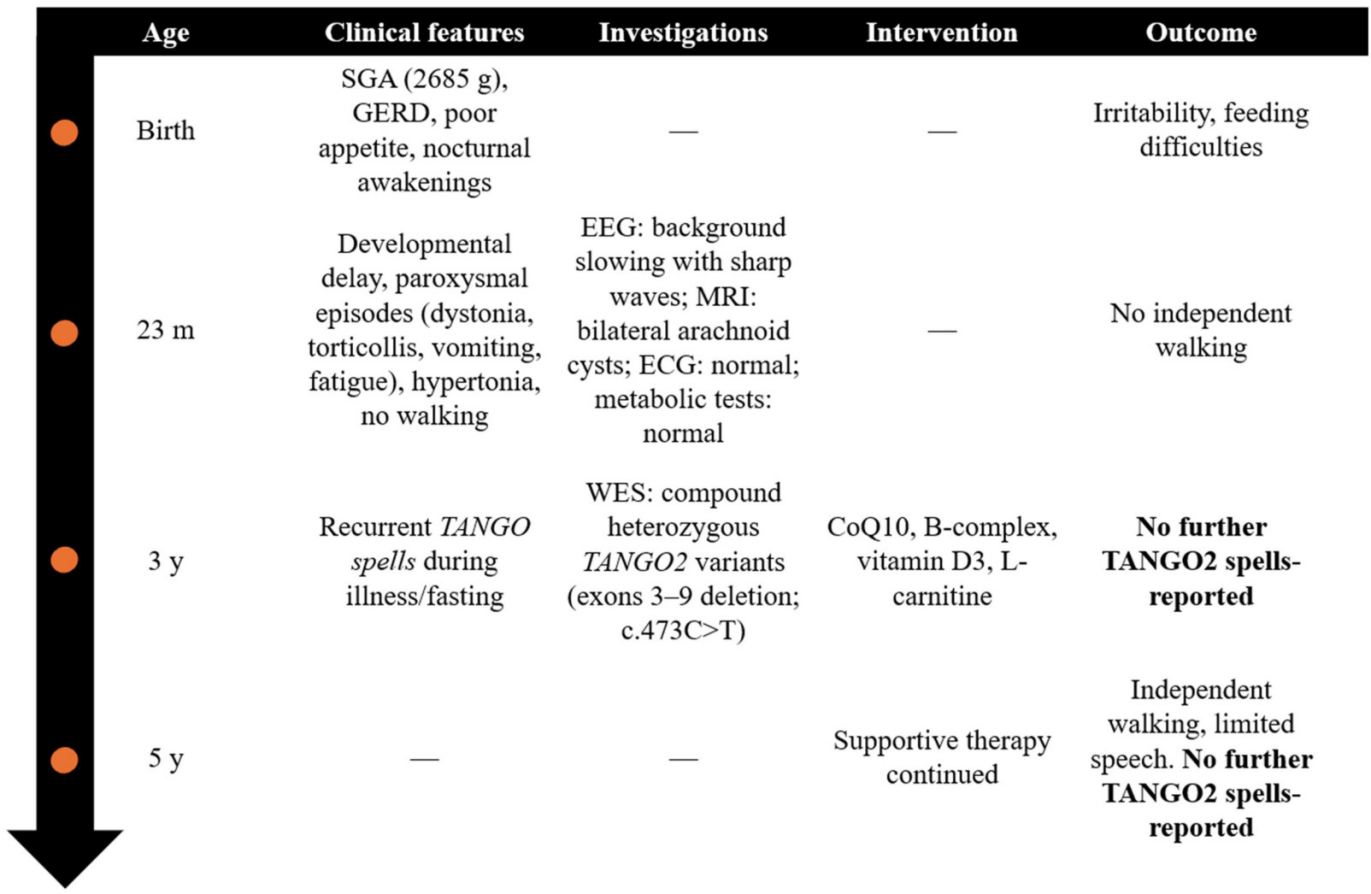
Timeline of clinical course, investigations, interventions, and outcomes in the reported patient with TANGO2 deficiency disorder. m, months; y, years; SGA, small for gestational age; GERD, gastro-esophageal reflux disease; WES, whole exome sequencing.

A timeline summarizing the clinical course, diagnostic investigations, therapeutic interventions, and outcomes is provided in Figure 1, while the clinical characteristics of TANGO2 spells in the reported patient are summarized in Table 1.

**Table 1:** Clinical characteristics of TANGO2 spells in the reported patient.

Feature	Description
Age at onset	23 months
Frequency	Recurrent, intermittent
Triggers	Illness, fasting
Semiology	Dystonia, torticollis, weakness, vomiting, reduced responsiveness
Duration	Several hours
EEG during spells	Non-ictal
Post-episode state	Prolonged fatigue
Metabolic abnormalities	Absent
Response to therapy	Resolution after supplementation

### 3.2 Results of the scoping review

**Figure 2:**
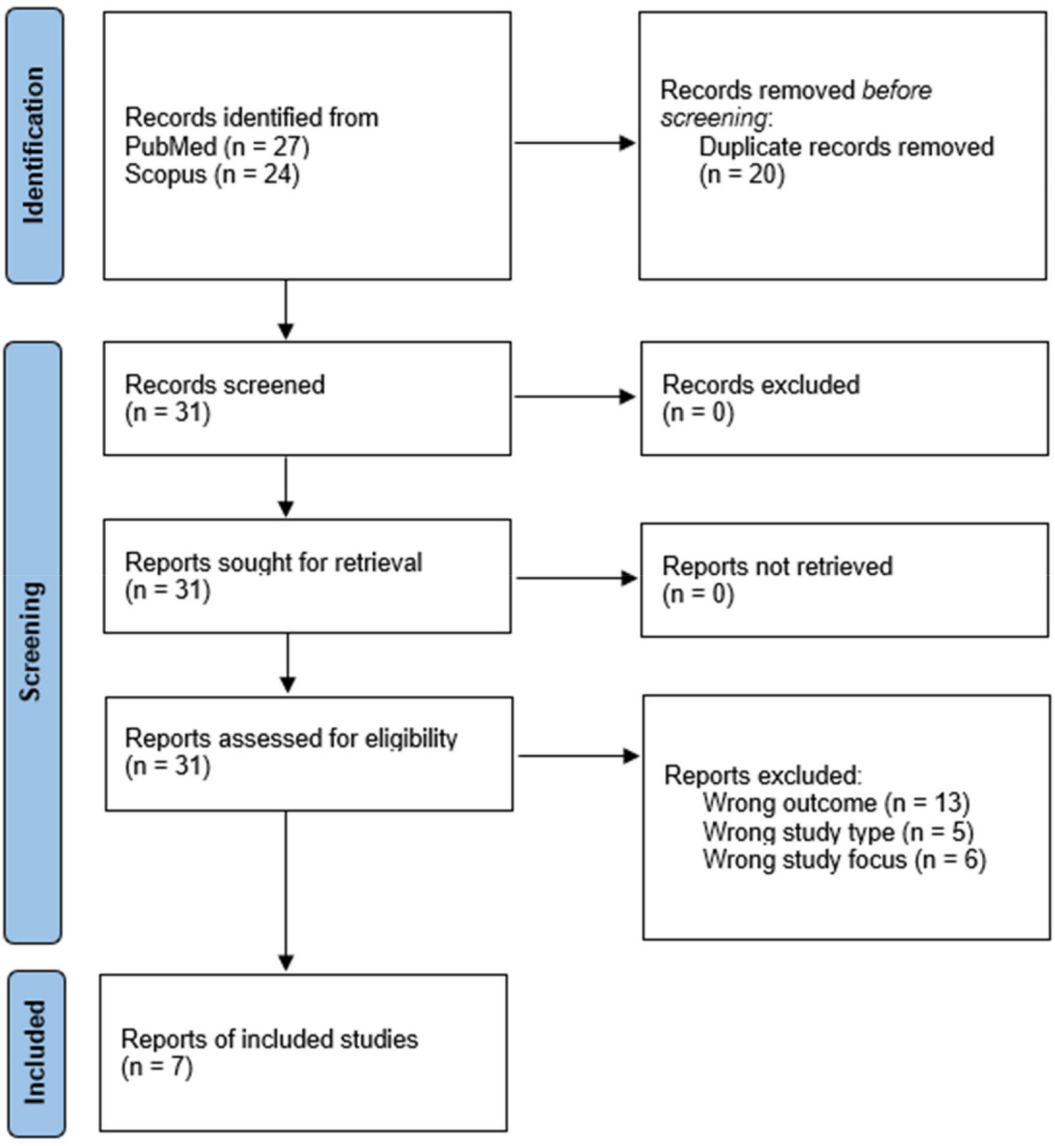
Flow-chart of study selection.

The literature search identified 31 records through PubMed and Scopus. After full-text assessment, 7 studies met the predefined eligibility criteria and were included in the scoping review. The study selection process is summarized in the PRISMA-ScR flow diagram (Figure 2).

The included studies comprised case reports, small case series, and one multicenter observational cohort, published between 2019 and 2025. Overall, the review encompassed a total of 93 patients with genetically confirmed TANGO2 deficiency disorder.

The largest contribution derived from the multicenter natural history study by Miyake et al. (7) (*n* = 73), in which TANGO2 spells were explicitly defined as transient, non-epileptic neurological episodes distinct from metabolic crises. Smaller cohorts included an 11-patient case series reported by Jennions et al. (9), where 6 patients experienced recurrent paroxysmal episodes without metabolic abnormalities, and an institutional case series by Powell et al. (10) (5 institutional patients), complemented by a broader literature review. Four additional single-patient case reports were identified (11–14).

Across studies, terminology used to describe these episodes was heterogeneous, including “TANGO2 spells” (7, 14), episodic ataxia or dystonia (10), alternating hemiplegia–like episodes (11), episodic dyskinesia (13) and nonspecific episodic weakness (14).

Age at onset was typically in early childhood, most frequently between infancy and 3 years of age (7, 12, 13), although later onset was reported in isolated cases (11). Episodes were characterized by transient worsening of baseline neurological function, including gait instability or inability to walk, dystonia, abnormal head or body tilt, weakness, dysarthria, drooling, lethargy and reduced responsiveness, with preserved consciousness in the majority of cases. Duration ranged from minutes to several hours, occasionally extending up to one day, and spontaneous resolution was consistently reported across all studies.

Triggering factors were commonly identified and included intercurrent illness, fasting or reduced oral intake, physical exertion, and exposure to cold (7, 11, 13, 14). In contrast, no consistent provoking factors were identified in the Jennions et al. (9) case series, highlighting interindividual variability.

Electroencephalographic findings during spells were rarely reported and, when available, were non-ictal, with normal or nonspecific interictal abnormalities (9, 13). No study documented electrographic seizures temporally associated with the paroxysmal episodes, supporting their non-epileptic nature.

Importantly, most studies emphasized the absence of metabolic imbalances during TANGO2 spells, particularly in cohorts where spells were explicitly differentiated from metabolic crises (7, 9). Cardiac involvement, including prolongation QT and malignant arrhythmias, was consistently reported only in association with metabolic crises, and not during isolated spells (10).

Acute management of spells was largely supportive, consisting of rest, sleep, and nutritional intake, with no standardized pharmacological interventions. Preventive strategies focused on avoidance of fasting and metabolic stress and on vitamin or cofactor supplementation, particularly B-complex vitamins. Several studies reported a reduction in spell frequency or complete resolution following initiation of supplementation (7, 12, 13, 15).

A detailed comparison of study characteristics, clinical features, triggers, diagnostic findings, and management strategies is provided in Table 2.

**Table 2:** Clinical and genetic features of reported TANGO2 spells across 7 studies included. For Powell et al. (10), only the five institutional patients with individual-level clinical data were included. Aggregated prevalence and genotype-frequency data from the accompanying literature review (total *n* = 76) were intentionally excluded.

Author, year	Study design/N patients	Terminology used	Genotype (variant class)	Age at onset of spells	Clinical semiology	Triggers	EEG during spells	Metabolic crisis associated	Cardiac involvement	Acute management	Preventive/long-term management	Outcome/follow-up
Sugiyama et al. (12)	Case report/*n* = 1	“TANGO2 spell”, transient ataxic episode	Compound heterozygous truncating variants (frameshift + intragenic deletion)	18 months	Sudden onset unsteadiness and inability to walk lasting hours–days; spontaneous resolution	Mild infections	Not assessed	Metabolic crises occurred later, temporally distinct from spells	None reported	None (spontaneous resolution)	Multivitamin supplementation incl. vitamin B5	No further spells or metabolic crises during 1-year follow-up; gradual developmental progress
Li et al. (13)	Case report/*n* = 1	Episodic dyskinesia; paroxysmal events	Compound heterozygous: nonsense (p.Gln218*) + missense (p.Gly235Ser)	6 months	Recurrent paroxysmal head deviation, dyskinesia, drooling, limb weakness or transient hemiparesis; preserved consciousness; resolution after rest/sleep	Illness, cold, fatigue	Ictal EEG not obtained; interictal epileptiform discharges	No metabolic crisis documented during spells; later coma after GTC seizure with unremarkable labs	No QT prolongation or structural abnormalities reported	Rest and sleep	High-dose vitamin B5 (for TDD) + oxcarbazepine (for epilepsy)	Marked reduction/cessation of paroxysmal events and developmental gains over ∼2 years
Miyake et al. (7)	Multicenter natural history study/*n* = 73	“TANGO2 spells” (explicitly defined)	Mixed: intragenic deletions (esp. exons 3–9), splice, nonsense, missense; no genotype–phenotype correlation	Median 18 months (IQR 12.5–24; range 4–36 months)	Transient ataxia or weakness, head tilt/retrocollis, lethargy, staring, slurred speech, drooling, paroxysmal dyskinesia without loss of consciousness; minutes–hours; spontaneous resolution	Warm weather, exertion, reduced intake/fasting, routine disruption, early morning; constipation	Not systematically assessed during spells	Absent during spells (defined by lack of laboratory evidence of metabolic decompensation), despite partial clinical overlap with crises	Absent during spells; arrhythmias only during metabolic crises	Rest, naps, snacks	B-complex or multivitamin supplementation (incl. B5/B9)	Spells may recur episodically; metabolic crises markedly reduced after supplementation
Sen et al. (11)	Case report/*n* = 1	AHC-like paroxysmal episodes	Homozygous splice-site variant	∼5 years	Alternating unilateral hemiplegia with dystonia; hours–1 day; preserved consciousness; resolution after sleep	Infections, cold exposure	Not assessed	Single episode of rhabdomyolysis at age 7, temporally distinct from hemiplegic episodes	None reported (normal ECG/echo)	Supportive care, sleep	Antiseizure medications; flunarizine trial (transient benefit)	Episodic symptoms persisted into adolescence
Skocy et al. (14)	Case report/*n* = 1	Episodic weakness	Compound heterozygous: exon 3–9 deletion + missense	Childhood (retrospective)	Recurrent episodes of bilateral lower limb weakness and fatigue; later episode with slurred speech	Respiratory infections	Not assessed	Yes, during later hospitalization with rhabdomyolysis	Marked QT prolongation during metabolic crisis	Inpatient supportive management	B-complex supplementation incl. high-dose B5	Episodic weakness reported lifelong; limited post-diagnosis follow-up
Jennions et al. (9)	Case series/11 patients (6 with spells)	Recurrent episodes of decreased consciousness; episodic weakness; ataxia (not formally labeled as “TANGO2 spells”)	Bi-allelic TANGO2 variants: exon 3–9 deletions, exon 4–6 deletions, frameshift, splice-site and missense variants (homozygous or compound heterozygous)	Childhood; variable onset (often preceding metabolic crises; exact ages inconsistently reported)	Recurrent paroxysmal episodes of decreased responsiveness, asymmetrical weakness or hemiparesis, leg pain and ataxia; preserved or fluctuating consciousness; duration 10 min to several hours; spontaneous resolution	None consistently identified	Video-EEG normal during episodes in one patient	No metabolic derangements documented during spells in investigated patients (≥3); spells could occur independently of metabolic crises	Absent during spells; arrhythmias during metabolic crises	Supportive care	Carbohydrate-rich diet; avoidance of fasting; vitamin/cofactor supplementation	Spells recurred intermittently; morbidity related to metabolic crises
Powell et al. (10)	Single-center retrospective case series + review/5 institutional patients (76 reviewed)	Episodic ataxia, dystonia, paroxysmal torticollis, alternating hemiplegia	Exons 3–9 deletion + 22q11.2 deletion (*n* = 1)	Infancy to early childhood	Paroxysmal ataxia, dystonia, gait disturbance, torticollis, hemiplegia	Illness, fasting, exertion, metabolic stress	Not systematically reported	Yes, in 4/5 patients during disease course; spells often preceded or occurred independently from crises	Yes, in 3/5 patients (QT prolongation, ventricular tachyarrhythmias, cardiomyopathy); 1 heart transplant	Supportive care during metabolic decompensation; ICU-level care during cardiac crises; no spell-specific acute therapy	Avoidance of fasting; carbohydrate-rich diet; vitamin/cofactor supplementation; cardiac surveillance	Variable; significant morbidity and mortality related to crises
			c.35_36delCT (homozygous frameshift) (*n* = 2 siblings)									
			Exons 4–6 deletion + c.451 + 2T > A splice-site variant (compound heterozygous) (*n* = 2 siblings)									

## 4 Discussion

In this study, we describe a child with TANGO2 deficiency disorder (TDD) presenting with recurrent paroxysmal non-epileptic neurological episodes and contextualize these findings within a scoping review of the literature. The close alignment between our case and previously reported cohorts supports the recognition of “TANGO2 spells” as a distinctive clinical phenomenon within the TDD spectrum.

The age at onset in our patient (23 months) is consistent with the early childhood presentation reported across studies, particularly in the multicenter cohort by Miyake et al. (7), where spells most frequently began before 3 years of age. As described in the literature, episodes in our patient were stress-related, prolonged, self-limited, and followed by marked fatigue (7, 9, 12). The observed semiology—weakness, dystonia with torticollis, vomiting, irritability, and reduced responsiveness without loss of consciousness—falls well within the range of manifestations reported in case series and single-patient reports (10, 13).

From a diagnostic standpoint, our case highlights the importance of careful electroclinical interpretation. In our patient, EEG recordings were obtained in the interictal state and showed mildly abnormal but nonspecific findings, without evidence of epileptiform activity. Although EEG monitoring was performed, no habitual events were captured, and no interictal epileptiform abnormalities were detected. The absence of electroclinical seizure correlates, together with the prolonged and stereotyped semiology, supported a non-epileptic classification. This is in line with available literature, where EEGs performed during or around spells were non-ictal when assessed (9, 13), and no study has documented electrographic seizures temporally associated with TANGO2 spells.

Differentiating TANGO2-related paroxysmal spells from epileptic seizures represents a key clinical challenge, particularly because epilepsy frequently coexists in patients with TANGO2 deficiency disorder (1, 7). TANGO2 spells may clinically resemble seizures; however, they typically occur in temporal association with physiological stressors such as intercurrent illness, fasting, or reduced intake. Importantly, when EEG or video-EEG monitoring has been performed during these episodes, ictal epileptiform activity has not been documented, with recordings showing normal or nonspecific findings (7, 9, 13). In contrast, epileptic seizures in TDD are usually brief, stereotyped, and supported by electroclinical correlates. Recognizing this distinction is clinically relevant, as TANGO2 spells primarily benefit from supportive and preventive metabolic strategies rather than escalation of antiseizure treatment in the absence of confirmed epilepsy (9, 10).

Similarly, no metabolic abnormalities were detected during spells in our patient, reinforcing the distinction between TANGO2 spells and metabolic crises emphasized in several reports (7, 9). Cardiac assessment was unremarkable, in keeping with evidence that QT prolongation and malignant arrhythmias are generally observed during metabolic crises rather than isolated spells (10).

A further contribution of our case is the standardized assessment of adaptive functioning using the Vineland Adaptive Behavior Scales, Second Edition (Vineland-II). While global developmental delay and intellectual disability are frequently reported in TDD cohorts (9, 10), detailed adaptive profiles are rarely provided. Our patient showed clinically significant adaptive impairments across multiple domains, with relative strengths in domestic daily living skills and gross motor abilities. Importantly, these deficits were present despite the absence of metabolic crises, supporting the hypothesis that neurodevelopmental dysfunction in TDD may reflect a primary disease component rather than solely crisis-related injury.

From a genetic perspective, our patient harbored compound heterozygous truncating variants, a genotype commonly observed in TDD (1, 2, 10). Consistent with prior studies, no clear genotype–phenotype correlation can be established with respect to the occurrence or severity of TANGO2 spells (7–9), suggesting that additional modifiers—developmental or environmental— are more likely to contribute to phenotypic variability.

Several reports have described a reduction in frequency or resolution of TANGO2-related paroxysmal episodes following the initiation of supportive metabolic interventions, including avoidance of fasting, carbohydrate-rich nutrition, prompt treatment of intercurrent illnesses, and supplementation with B-complex vitamins, particularly pantothenic acid (7, 9, 12, 13). In the largest natural history cohort, vitamin and cofactor supplementation was associated with a marked reduction in metabolic crises and was frequently accompanied by a decrease in paroxysmal neurological episodes, although causality could not be established (7). Importantly, no standardized treatment protocols for TANGO2 spells currently exist, and reported improvements derive from uncontrolled observational data, often following the simultaneous initiation of multiple interventions (10, 13). Antiseizure medications have not consistently demonstrated efficacy in preventing TANGO2 spells in the absence of confirmed epilepsy, supporting a management approach focused on metabolic stability and trigger avoidance. Prospective studies with standardized outcome measures are needed to clarify the impact of supportive therapies on paroxysmal neurological manifestations in TANGO2 deficiency disorder.

In our patient, a reduction in spells followed the introduction of vitamin supplementation; however, given the natural variability of the disorder and the absence of controlled data, a causal relationship cannot be established. These observations highlight the need for prospective, systematically collected outcome data in TANGO2 deficiency disorder.

## 5 Conclusions

TANGO2 spells represent a distinctive, early, non-epileptic neurological manifestation of TANGO2 deficiency disorder that may occur independently of metabolic decompensation. Our case closely mirrors the phenotype described in the literature and adds novel detail through standardized adaptive assessment.

Recognition of these spells is clinically relevant, as they may precede metabolic crises and facilitate earlier diagnosis and anticipatory management. Future studies should focus on refining diagnostic criteria, clarifying modifiers of phenotypic expression beyond genotype, and generating prospective evidence to guide management strategies in this rare disorder.

## Data Availability

The raw data supporting the conclusions of this article will be made available by the authors, without undue reservation.

## Supplementary material

Supplementary Video 1 Representative episodes of TANGO2 spells in the reported patient.

Supplementary Figure S1 Interictal EEG showing background slowing with superimposed fast rhythms and occasional sharp waves.

Supplementary Figure S2 Brain MRI showing bilateral temporopolar arachnoid cysts with ventricular dilation and enlarged CSF spaces.
