# Supplementary figures and images for: Paroxysmal neurological spells in TANGO2 deficiency disorder: a case report and a scoping review
Source: Front Pediatr. 2026 Apr 2;14:1786640. doi: 10.3389/fped.2026.1786640 (PMC13085313; doi:10.3389/fped.2026.1786640)

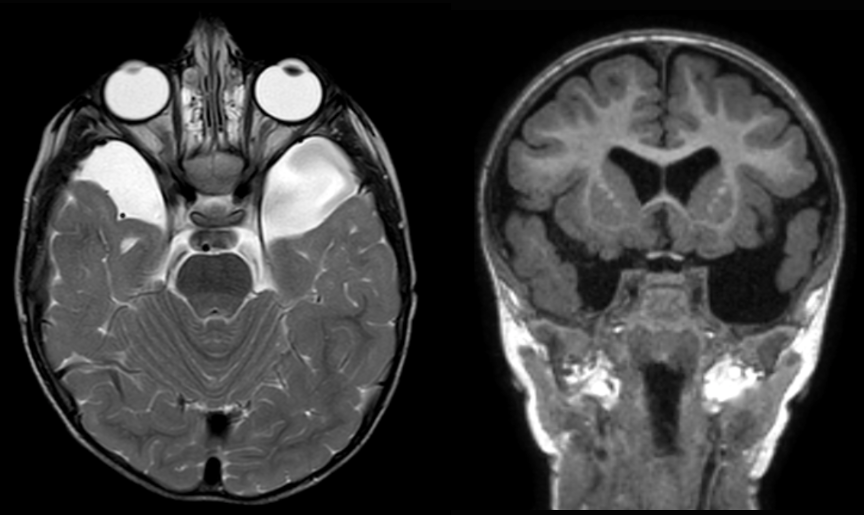

Supplement: Supplementary Figure S1 — Interictal EEG showing background slowing with superimposed fast rhythms and occasional sharp waves. [file Image1.png]
