# Supplementary material for: Paroxysmal neurological spells in TANGO2 deficiency disorder: a case report and a scoping review
Source: Front Pediatr. 2026 Apr 2;14:1786640. doi: 10.3389/fped.2026.1786640 (PMC13085313; doi:10.3389/fped.2026.1786640)
Supplement: Supplementary Figure S2 — Brain MRI showing bilateral temporopolar arachnoid cysts with ventricular dilation and enlarged CSF spaces. [file Image2.pdf]

Age	Clinical features	Investigations	Intervention	Outcome
Birth	SGA (2685 g), GERD, poor appetite, nocturnal awakenings	—	—	Irritability, feeding difficulties
23 m	Developmental delay, paroxysmal episodes (dystonia, torticollis, vomiting, fatigue), hypertonia, no walking	EEG: background slowing with sharp waves; MRI: bilateral arachnoid cysts; ECG: normal; metabolic tests: normal	—	No independent walking
3 y	Recurrent *TANGO spells* during illness/fasting	WES: compound heterozygous *TANGO2* variants (exons 3–9 deletion; c.473C>T)	CoQ10, B-complex, vitamin D3, L-carnitine	**No further TANGO2 spells-reported**
5 y	—	—	Supportive therapy continued	Independent walking, limited speech. **No further TANGO2 spells-reported**
